# ChagasDB: 80 years of publicly available data on the molecular host response to *Trypanosoma cruzi* infection in a single database

**DOI:** 10.1093/database/baad037

**Published:** 2023-05-26

**Authors:** Pauline Brochet, Jean-Christophe Mouren, Laurent Hannouche, Fabrice Lopez, Benoit Ballester, Edecio Cunha-Neto, Lionel Spinelli, Christophe Chevillard

**Affiliations:** Institut National de la Santé Et de la Recherche Médicale (INSERM), Unité Mixte de Recherche (UMR)_1090, Aix Marseille Université, TAGC Theories and Approaches of Genomic Complexity, Institut MarMaRa, Marseille 13288, France; Institut National de la Santé Et de la Recherche Médicale (INSERM), Unité Mixte de Recherche (UMR)_1090, Aix Marseille Université, TAGC Theories and Approaches of Genomic Complexity, Institut MarMaRa, Marseille 13288, France; C2VN, INSERM, INRA, Aix Marseille Université, Marseille 13005, France; Institut National de la Santé Et de la Recherche Médicale (INSERM), Unité Mixte de Recherche (UMR)_1090, Aix Marseille Université, TAGC Theories and Approaches of Genomic Complexity, Institut MarMaRa, Marseille 13288, France; Institut National de la Santé Et de la Recherche Médicale (INSERM), Unité Mixte de Recherche (UMR)_1090, Aix Marseille Université, TAGC Theories and Approaches of Genomic Complexity, Institut MarMaRa, Marseille 13288, France; Laboratory of Immunology, Heart Institute Instituto do Coração (InCor), University of São Paulo, School of Medicine, São Paulo 05403-900, Brazil; Division of Clinical Immunology and Allergy, University of São Paulo, School of Medicine, São Paulo 05403-900, Brazil; Instituto Nacional de Ciência e Tecnologia, INCT, III- Institute for Investigation in Immunology, São Paulo 05403-900, Brazil; Institut National de la Santé Et de la Recherche Médicale (INSERM), Unité Mixte de Recherche (UMR)_1090, Aix Marseille Université, TAGC Theories and Approaches of Genomic Complexity, Institut MarMaRa, Marseille 13288, France; Institut National de la Santé Et de la Recherche Médicale (INSERM), Unité Mixte de Recherche (UMR)_1090, Aix Marseille Université, TAGC Theories and Approaches of Genomic Complexity, Institut MarMaRa, Marseille 13288, France

## Abstract

Chagas disease is a parasitical disease caused by *Trypanosoma cruzi* which affects ∼7 million people worldwide. Per year, ∼10 000 people die from this pathology. Indeed, ∼30% of humans develop severe chronic forms, including cardiac, digestive or neurological disorders, for which there is still no treatment. In order to facilitate research on Chagas disease, a manual curation of all papers corresponding to ‘Chagas disease’ referenced on PubMed has been performed. All deregulated molecules in hosts (all mammals, humans, mice or others) following *T. cruzi* infection were retrieved and included in a database, named ChagasDB. A website has been developed to make this database accessible to all. In this article, we detail the construction of this database, its contents and how to use it.

**Database URL** https://chagasdb.tagc.univ-amu.fr

## Introduction

Chagas disease is a parasitical disease caused by a protozoan, *Trypanosoma cruzi*. This parasite is endemic from South America and contaminates multiple mammal species through a vector of the *Reduviidae* family or indirect contamination (food contaminated, congenital transmission…) (1). After infection with *T. cruzi*, infected mammals first present an acute phase. This phase is mostly asymptomatic but sometimes leads to physical signs, such as Romaña’s sign, as well as fever or headache. During this phase, a strong immune response is set up, characterized by an overproduction of Th1 lymphocytes, which will produce interferon gamma and destroy the parasite, albeit not providing sterilizing immunity (2). After several weeks, the infected mammals enter the chronic phase, which is mostly asymptomatic. However, severe forms can develop, notably cardiac, digestive, cardiodigestive or nervous. In humans, ∼30% of individuals in the chronic phase will develop these complications, the most frequent being chronic Chagas cardiomyopathy. Chagas disease affects ∼7 million of people, and per year, ∼10 000 people die from this pathology (3).

To understand the development of Chagas disease and to find effective treatments, many research teams have worked not only on patient samples but also on animal models (4, 5), such as mice, rats, dogs, hamsters or even sheep. Over the years, these studies have revealed numerous deregulated molecules in the hosts, associated with the infection of the parasite. With the advancement of technology, increasing number of features has been characterized, like mutations, genes, proteins, hormones or other chemical molecules. However, there is currently no database containing all this information. It is therefore difficult to make the links between new results and what has already been identified by the community.

To facilitate the sharing of knowledge related to Chagas disease and thus research on this disease, we have reviewed 193 articles from 1945 to 2022 and collected information on molecules in hosts associated with *T. cruzi* infection. The collected information has been summarized in a freely accessible online database, ChagasDB (https://chagasdb.tagc.univ-amu.fr), and as a downloadable file. Thus, any molecule of interest can easily be found, regardless of the organism, its type or the strain of *T. cruzi* responsible for its dysregulation. These data can then be used to enrich other studies. In this article, we detail the construction criteria of this database, as well as its content and how to use it.

## Materials and methods

### Article review

We selected the article matching the search ‘Chagas disease’ in PubMed. The selection of the articles was made following several steps based on different criteria. In the first time, the title of each article was read, and all articles not related to research on host responses to *T. cruzi* infection were discarded. Therefore, any review, epidemiological analysis or study of the parasite or insect vector has not been included in ChagasDB. The second step of article selection was performed based on the abstract. Only unmodified hosts (not knocked out or stimulated with a particular chemical compound) were retained. This can be *in vivo* studies or *in vitro* studies with unmodified cells. Finally, the third and last steps were applied, reading the ‘Material and Methods’ section, to verify that the article meets the established criteria. When selected, the complete article was parsed to review the results. Analyses showing significant associations between *T. cruzi* infection and host molecules were included in the database.

For each molecule of interest, the following information was retrieved: its name as used in the article, its type, its level of variation, the compared phenotypes, the analyzed tissue, the method of analysis, the organism, the strain of *T. cruzi* (if known), the Digital Object Identifier (DOI) and PubMed reference number (PMID) of the article, its title and its year of publication.

### Database annotation

The collected features were annotated using different reference databases, depending on the species. For all genes-related features (protein, methylation site, mutation…), the name, description and ID of the associated genes were reported. In that case, for all of them, Ensembl (6) database was used. For *Homo sapiens* specifically, HUGO Gene Nomenclature Committee (7) and GeneCards (8) helped to identify some alias. microRNADataBase (miRBase) (9) and LNCipedia (10) were used for noncoding RNA. The Mouse Genome Informatics (11) and Rat Genome Database (12) were employed for *Mus musculus* and *Rattus norvegicus*, respectively.

### Website development

The platform has been developed using a Python (v3.9.13) environment with the Django (v.4.0.7) framework coupled to a SQLite (v3.39.3) database. The web interface makes use of Jquery (v3.6.0), HTML5 and the framework TailwindCSS (v3.2.4). ChagasDB was deployed with Docker (v20.10.12) and Docker Compose (v1.25.0). A CLI (command–line interface) tool using Typer (v0.4.1) was developed to ensure an easy and fast update of the platform. All the scripts used to develop the website are available at https://github.com/TAGC-ComplexDisease/ChagasDB.

### ChagasDB features analysis

To get an overview of the information contained in the ChagasDB, a functional enrichment analysis was applied using R language. Due to the low number of features identified in *R. norvegicus*, *Canis lupus familiaris* and *Ovis aries*, only human and mouse features were considered for Gene Ontology (GO) biological process enrichment. Deregulated genes and proteins were analyzed using gProfileR (v0.7.0) (13), and top GOs were recovered using rrvigo (v1.8.0) (14). Finally, the results were illustrated using ggplot2. To compare genes or proteins between different species, orthologous genes were found using biomaRt R package (v2.52.0) (15).

## Results and discussion

**Figure 1.**
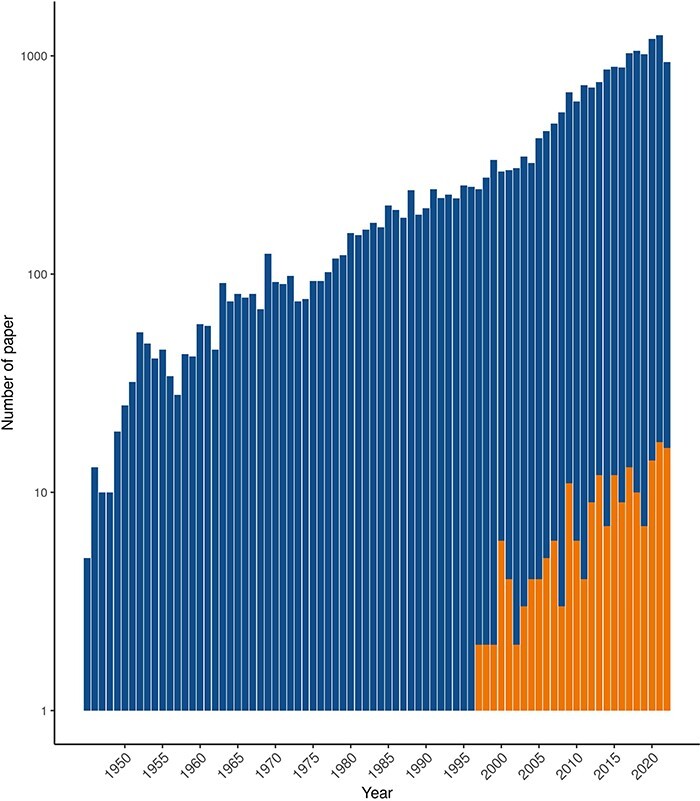
Number of paper references as ‘Chagas disease’ in PubMed (blue) and included in ChagasDB (orange) per year.

Among the 21 121 articles listed in PubMed as ‘Chagas disease’, we have retained 193 articles (Figure 1) that correspond to our selection criteria: experimental or computational analysis that found significant deregulation at molecular levels on host infected by *T. cruzi*. Although the oldest paper referenced in PubMed dates from 1945, the 193 articles in ChagasDB were published between 1995 and 2022, due to the lack of statistical test before 1995 (Supplementary Table S1). Information for each feature in the database was gathered, comprising a set of 25 distinct categories. These categories included details related to the feature itself, such as its name, description, ID and type, as well as information regarding the study conducted, including tissue, phenotypes, analysis, organism, population and *T. cruzi* strain. Additionally, the paper’s DOI, PMID, title and publication year were also included in the information set. All the details are available on Supplementary Table S2.

For now, 19 phenotypes are available in ChagasDB: five as *T. cruzi*–uninfected control (with several phenotypes) and 14 as cases, with different forms of Chagas disease. The controls comprise healthy controls and non-chagasic cardiomyopathy, including dilated, idiopathic and ischemic cardiomyopathy. In *T. cruzi*–infected phenotype, all disease stages are available, including acute stage, asymptomatic chronic stage and symptomatic chronic stage: cardiac (moderate, mild, severe or all), digestive, cardiodigestive or mixed. Some congenital infection and heart transplant chagasic patients were also included. Concerning the parasite, 24 *T. cruzi* strains were included in the database, corresponding to three discrete typing units, TcI, TcII and TcVI. For TcI were used Colombia, G, Sylvio, SylvioX10/4, AC, CA-I, Col1.8G2, K98 and Type 1 strains. TcII include ABC, Berenice-78, Y, JG, Lucky and SGO-Z12 strains. Finally, TcVI comprise CL-14, CL-Brener, Cvd, Q501/3, Tulahuén, Tehuantepec, RA and VD strains.

From the 193 articles in ChagasDB, 174 present experimental analysis of molecules associated with *T. cruzi* infection, while 33 present computational analysis. Most of them were interested in *H. sapiens* response to *T. cruzi* (142), but some animal models were also employed: *M. musculus* (40), *R. norvegicus* (12), *O. aries* (1) and *C. lupus familiaris* (1). As a result, a greater number and diversity of molecules have been identified in humans compared to other mammals (Table 1).

**Table 1.** Number of features included in ChagasDB by species

	*Homo sapiens*	*Mus musculus*	*Rattus norvegicus*	*Canis lupus familiaris*	*Ovis aries*
RNA	10 534	5885	289	11	3
Alternative splicing	1778				
Methylation	41 041				
Transcription factor binding site (TFBS)	30				
Polymorphism	49				
Haplogroup	6				
Protein	339	56	15	3	
Protein activity	7	7			
Chemical molecule	49	2	5		
Electrolyte	1	2			
Hormone	8				

**Figure 2.**
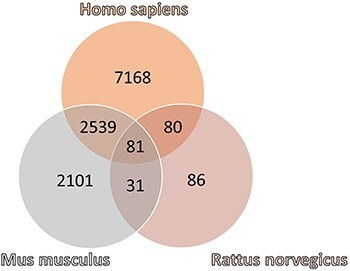
Venn diagram of the number of genes and proteins referenced in ChagasDB in *Homo sapiens, Mus musculus* and *Rattus norvegicus.*

However, a large percent of genes and proteins deregulated are shared between mammals (Figure 2), with 55% of mouse, 69% of rat and 27% of human features shared between at least two species.

**Figure 3.**
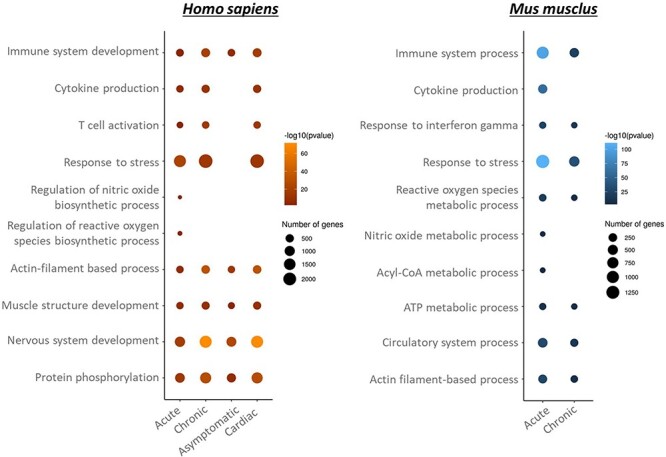
GO biological process enrichment of genes and proteins deregulated in *Homo sapiens* and *Mus musculus* per disease stage.

Beyond the deregulated genes shared between humans and mice, similar biological functions are affected at different stages of the disease (Figure 3). In both species, the immune response and muscle-related process are affected in all disease stages, whereas nitric oxide process seems to be specific to acute stage. Interestingly, in *H. sapiens*, the immune response is not deregulated in asymptomatic stage, unlike acute and chronic symptomatic stages. Due to the low number of features associated with other phenotypes (digestive, cardiodigestive…), these phenotypes were not taken into account in the analysis (Supplementary Table S3).

While acute and asymptomatic forms of studies represent, respectively, 51 (26%) and 77 (40%) articles, cardiac forms totalize 141 articles (73%) of the whole 193 articles, illustrating the interest for this specific disease presentation. There is an urgent need to understand why it develops in order to find a treatment. The development of databases makes it possible to gather a large amount of information that is directly and easily accessible, thus facilitating research. Moreover, the pooling of results, which are sometimes difficult to access, makes it possible to at least confirm one’s own results and at best save time and resources. For this purpose, we have developed a website allowing not only to explore ChagasDB though queries but also to download the whole database. Briefly, users can enter a feature name, ID or description in the ‘Search’ bar tool, and the different corresponding features will appear. They can download the information relative to the features of interest. Users can apply some filters on different fields (like organism, *T. cruzi* strain…) to have access to a restricted number of results that correspond to their needs. Finally, users can also submit their own research results to improve the ChagasDB content.

To conclude, ChagasDB is a database gathering all the molecules identified in the literature as deregulated in hosts due to *T. cruzi* infection. These data are easily accessible via a website, to be integrated in different analyses, or to be used as a confirmation. To our knowledge, this is the first database related to molecular response to Chagas disease. Currently, ChagasDB is limited to host molecules, but in the future, it could be extended to other types of data: cellular deregulations, responses to stimuli or treatments. Its objective will always be to facilitate research on Chagas disease, in order to help achieve a complete cure.

## Supplementary Material

baad037_Supp

## Data Availability

The ChagasDB can be downloaded by everybody without preliminary authorization.

## Feedback

The ChagasDB team thanks our future users for their future feedback to make ChagasDB useful for the community. Please contact for development requests (pauline.brochet@gmail.com or christophe.chevillard@univ-amu.fr).

## References

[1] Pérez-Molina J.A. and Molina I. (2018) Chagas disease. *Lancet*, 391, 82–94. 28673423 10.1016/S0140-6736(17)31612-4

[2] Cristovão-Silva A.C. , Brelaz-de-castro M.C.A., Hernandes M.Z. et al. (2021) Chagas disease: Immunology of the disease at a glance. *Cytokine Growth Factor Rev.*, 62, 15–22. 34696979 10.1016/j.cytogfr.2021.10.001

[3] Lee B.Y. , Bacon K.M., Bottazzi M.E. et al. (2013) Global economic burden of Chagas disease: a computational simulation model. *Lancet Infect Dis*, 13, 342–348. 23395248 10.1016/S1473-3099(13)70002-1 PMC3763184

[4] Scharfstein J. , Gomes J.D.A.S. and Correa-Oliveira R. (2009) Back to the future in Chagas disease: from animal models to patient cohort studies, progress in immunopathogenesis research. *Mem. Inst. Oswaldo Cruz*, 104 Suppl 1, 187–198. 19753474 10.1590/s0074-02762009000900025

[5] Chatelain E. and Scandale I. (2020) Animal models of Chagas disease and their translational value to drug development. *Expert Opin. Drug Discov.*, 15, 1381–1402. 32812830 10.1080/17460441.2020.1806233

[6] Cunningham F. , Allen J.E., Allen J. et al. (2022) Ensembl 2022. *Nucleic Acids Res.*, 50, D988–D995. 34791404 10.1093/nar/gkab1049 PMC8728283

[7] Tweedie S. , Braschi B., Gray K. et al. (2020) Genenames.org: the HGNC and VGNC resources in 2021. *Nucleic Acids Res.*, 49, D939–D946. 10.1093/nar/gkaa980 PMC7779007 33152070

[8] Stelzer G. , Rosen R., Plaschkes I. et al. (2016) The GeneCards Suite: From Gene Data Mining to Disease Genome Sequence Analyses. *Current Protocols in Bioinformatics*, 54, 1–30. 10.1002/cpbi.5 27322403

[9] Kozomara A. , Birgaoanu M. and Griffiths-Jones S. (2019) miRBase: from microRNA sequences to function. *Nucleic Acids Res.*, 47, D155–D162. 30423142 10.1093/nar/gky1141 PMC6323917

[10] Volders P.-J. , Anckaert J., Verheggen K. et al. (2019) LNCipedia 5: towards a reference set of human long non-coding RNAs. *Nucleic Acids Res.*, 47, D135–D139. 30371849 10.1093/nar/gky1031 PMC6323963

[11] Blake J.A. , Baldarelli R., Kadin J.A. et al. (2020) Mouse Genome Database (MGD): knowledgebase for mouse–human comparative biology. *Nucleic Acids Res.*, 49, D981–D987. 10.1093/nar/gkaa1083 PMC7779030 33231642

[12] Smith J.R. , Hayman G.T., Wang S.-J. et al. (2020) The Year of the Rat: the Rat Genome Database at 20: a multi-species knowledgebase and analysis platform. *Nucleic Acids Res.*, 48, D731–D742. 31713623 10.1093/nar/gkz1041 PMC7145519

[13] Reimand J. , Arak T., Adler P. et al. (2016) g:Profiler-a web server for functional interpretation of gene lists (2016 update). *Nucleic Acids Res.*, 44, W83–W89. 27098042 10.1093/nar/gkw199 PMC4987867

[14] Sayols S. (2023) rrvgo: a Bioconductor package for interpreting lists of Gene Ontology terms. *MicroPubl. Biol.*, 2023, doi: 10.17912/micropub.biology.000811. PMC10155054 37151216

[15] Durinck S. , Spellman P.T., Birney E. et al. (2009) Mapping identifiers for the integration of genomic datasets with the R/Bioconductor package biomaRt. *Nat. Protoc.*, 4, 1184–1191. 19617889 10.1038/nprot.2009.97 PMC3159387

