# Supplementary material for: ChagasDB: 80 years of publicly available data on the molecular host response to Trypanosoma cruzi infection in a single database
Source: Database (Oxford). 2023 May 26;2023:baad037. doi: 10.1093/database/baad037 (PMC10205463; doi:10.1093/database/baad037)
Supplement: baad037_Supp [file baad037_supp.zip › suppl_data/Supplementary_Table 1.docx]

**Supplementary table 1.** Description of ChagasDB field.

| Column name | Description |
| --- | --- |
| Feature name | Feature official name according to databases. |
| Feature description | Description about the features which provide some details according to databases.  In some case, no description is available. |
| Feature name in paper | Feature name provided by authors. May be different to the official name. |
| Feature ID | Feature ID according to databases. It may be Ensembl ID for genes or proteins, cg ID for methylation site or rs ID for polymorphisms.  In some case, no ID is available. |
| Feature type | Type of the feature in the relative paper. |
| Feature variation | Some additional information about the analysis performed on the relative feature. It may be a fold change, or others kind of dysregulation. |
| Phenotype 1 | Host phenotype associated with the relative feature dysregulation. |
| Description phenotype 1 | Additional information on the phenotype of interest. |
| Phenotype 2 | Host phenotype used as control in the relative study. It may be identical to Phenotype 1 when the analysis was focus on association between two features. |
| Description phenotype 2 | Additional information on the control phenotype. |
| Tissue | Tissue or cells in which the feature of interest was studied. |
| Tissue description | Additional information on the tissue when provided. |
| Experimental | Indicate if the analysis is experimental or computational. |
| Method | Name or type of method employed to analyze the feature of interest. |
| Interaction observed | Indicate if an interaction/association was observed between the feature of interest and another feature. |
| Name of the interactive feature | If an interaction is observed between the feature of interest and another feature, indicate its name. |
| ID of the interactive feature | If an interaction is observed between the feature of interest and another feature, indicate its ID. |
| Type of the interactive feature | If an interaction is observed between the feature of interest and another feature, indicate its type. |
| Organism | Name of the species in which the analysis was carried out. |
| Population | Additional information on the population of interest. |
| *T. cruzi* strain | If known, provide the strain of *T.cruzi* having infected the host. |
| Paper PMID | Provide the paper Pubmed ID. |
| Paper DOI | Provide the paper DOI. |
| Paper title | Provide the paper title. |
| Paper publication date | Provide the paper publication date. |
