# Supplementary material for: ChagasDB: 80 years of publicly available data on the molecular host response to Trypanosoma cruzi infection in a single database
Source: Database (Oxford). 2023 May 26;2023:baad037. doi: 10.1093/database/baad037 (PMC10205463; doi:10.1093/database/baad037)
Supplement: baad037_Supp [file baad037_supp.zip › suppl_data/Supplementary_Table 2.docx]

**Supplementary table 2.** List of the original articles included in the database.

| **Paper DOI** | **Paper reference** |
| --- | --- |
| 10.1016/0167-5273(95)02459-A | Davila DF, Bellabarba G, Hernandez L, Calmon G, Torres A, Donis JH, et al. Plasma norepinephrine, myocardial damage and left ventricular systolic function in Chagas’ heart disease. Int J Cardiol. 24 nov 1995;52(2):145‑51. |
| 10.1006/bbrc.1996.0900 | Chandrasekar B, Melby PC, Troyer DA, Freeman GL. Induction of proinflammatory cytokine expression in experimental acute Chagasic cardiomyopathy. Biochem Biophys Res Commun. 14 juin 1996;223(2):365‑71. |
| 10.1046/j.1365-3083.1997.d01-362.x | Dutra WO, Gollob KJ, Pinto-Dias JC, Gazzinelli G, Correa-Oliveira R, Coffman RL, et al. Cytokine mRNA profile of peripheral blood mononuclear cells isolated from individuals with *Trypanosoma cruzi* chronic infection. Scand J Immunol. janv 1997;45(1):74‑80. |
| 10.1002/clc.4960201013 | Carrasco HA, Alarcon M, Olmos L, Burguera J, Burguera M, Dipaolo A, et al. Biochemical characterization of myocardial damage in chronic Chagas’ disease. Clin Cardiol. oct 1997;20(10):865‑9. |
|  | Chandrasekar B, Melby PC, Troyer DA, Colston JT, Freeman GL. Temporal expression of pro-inflammatory cytokines and inducible nitric oxide synthase in experimental acute Chagasic cardiomyopathy. Am J Pathol. avr 1998;152(4):925‑34. |
| 10.1016/s0165-2478(97)00167-3 | Chandrasekar B, Melby PC, Pennica D, Freeman GL. Overexpression of cardiotrophin-1 and gp130 during experimental acute Chagasic cardiomyopathy. Immunol Lett. avr 1998;61(2‑3):89‑95. |
| 10.1128/iai.67.8.4033-4040.1999 | Van Overtvelt L, Vanderheyde N, Verhasselt V, Ismaili J, De Vos L, Goldman M, et al. *Trypanosoma cruzi* infects human dendritic cells and prevents their maturation: inhibition of cytokines, HLA-DR, and costimulatory molecules. Infect Immun. août 1999;67(8):4033‑40. |
| 10.1128/iai.67.11.5579-5586.1999 | Truyens C, Torrico F, Lucas R, De Baetselier P, Buurman WA, Carlier Y. The endogenous balance of soluble tumor necrosis factor receptors and tumor necrosis factor modulates cachexia and mortality in mice acutely infected with *Trypanosoma cruzi*. Infect Immun. nov 1999;67(11):5579‑86. |
| 10.1016/s0165-2478(99)00172-8 | Antúnez MI, Cardoni RL. IL-12 and IFN-gamma production, and NK cell activity, in acute and chronic experimental *Trypanosoma cruzi* infections. Immunol Lett. 1 févr 2000;71(2):103‑9. |
| 10.1034/j.1399-0039.2000.550301.x | Nieto A, Beraún Y, Collado MD, Caballero A, Alonso A, González A, et al. HLA haplotypes are associated with differential susceptibility to *Trypanosoma cruzi* infection. Tissue Antigens. mars 2000;55(3):195‑8. |
| 10.1016/s1054-8807(00)00027-2 | Benvenuti LA, Higuchi ML, Reis MM. Upregulation of adhesion molecules and class I HLA in the myocardium of chronic chagasic cardiomyopathy and heart allograft rejection, but not in dilated cardiomyopathy. Cardiovasc Pathol. 2000;9(2):111‑7. |
| 10.1046/j.1365-2249.2000.01258.x | Chandrasekar B, Melby PC, Troyer DA, Freeman GL. Differential regulation of nitric oxide synthase isoforms in experimental acute chagasic cardiomyopathy. Clin Exp Immunol. juill 2000;121(1):112‑9. |
| 10.1016/s0198-8859(00)00161-0 | Layrisse Z, Fernandez MT, Montagnani S, Matos M, Balbas O, Herrera F, et al. HLA-C(*)03 is a risk factor for cardiomyopathy in Chagas disease. Hum Immunol. sept 2000;61(9):925‑9. |
| 10.1016/s1054-8807(00)00045-4 | Petkova SB, Tanowitz HB, Magazine HI, Factor SM, Chan J, Pestell RG, et al. Myocardial expression of endothelin-1 in murine *Trypanosoma cruzi* infection. Cardiovasc Pathol. 2000;9(5):257‑65. |
| 10.1046/j.1365-3083.2001.00916.x | Laucella SA, Riarte A, Prado N, Zapata J, Segura EL. α4 Integrins and Sialyl Lewis x Modulation in Chronic Chagas Disease: Further Evidence of Persistent Immune Activation. Scandinavian Journal of Immunology. 2001;53(5):514‑9. |
| 10.1016/s0002-9149(01)01502-8 | Salomone OA, Caeiro TF, Madoery RJ, Amuchástegui M, Omelinauk M, Juri D, et al. High plasma immunoreactive endothelin levels in patients with Chagas’ cardiomyopathy. Am J Cardiol. 15 mai 2001;87(10):1217‑20; A7. |
| 10.1016/s0165-2478(01)00283-8 | Antúnez MI, Cardoni RL. Early IFN-gamma production is related to the presence of interleukin (IL)-18 and the absence of IL-13 in experimental *Trypanosoma cruzi* infections. Immunol Lett. 3 déc 2001;79(3):189‑96. |
| 10.1034/j.1399-0039.2001.580302.x | Calzada J e., Nieto A, Beraún Y, Martín J. Chemokine receptor CCR5 polymorphisms and Chagas’ disease cardiomyopathy. Tissue Antigens. 2001;58(3):154‑8. |
| 10.4049/jimmunol.168.12.6366 | Ouaissi A, Guilvard E, Delneste Y, Caron G, Magistrelli G, Herbault N, et al. The *Trypanosoma cruzi* Tc52-released protein induces human dendritic cell maturation, signals via Toll-like receptor 2, and confers protection against lethal infection. J Immunol. 15 juin 2002;168(12):6366‑74. |
| 10.1086/345882 | Araújo-Jorge TC, Waghabi MC, Hasslocher-Moreno AM, Xavier SS, Higuchi M de L, Keramidas M, et al. Implication of transforming growth factor-beta1 in Chagas disease myocardiopathy. J Infect Dis. 15 déc 2002;186(12):1823‑8. |
| 10.1016/s0925-4439(03)00060-7 | Garg N, Popov VL, Papaconstantinou J. Profiling gene transcription reveals a deficiency of mitochondrial oxidative phosphorylation in *Trypanosoma cruzi*-infected murine hearts: implications in chagasic myocarditis development. Biochim Biophys Acta. 14 juill 2003;1638(2):106‑20. |
| 10.1128/IAI.71.8.4441-4447.2003 | Petersen CA, Burleigh BA. Role for interleukin-1 beta in *Trypanosoma cruzi*-induced cardiomyocyte hypertrophy. Infect Immun. août 2003;71(8):4441‑7. |
| 10.1590/s0074-02762003000300021 | Ferreira RC, Ianni BM, Abel LCJ, Buck P, Mady C, Kalil J, et al. Increased plasma levels of tumor necrosis factor-alpha in asymptomatic/"indeterminate" and Chagas disease cardiomyopathy patients. Mem Inst Oswaldo Cruz. avr 2003;98(3):407‑11. |
| 10.1086/380803 | Talvani A, Rocha MOC, Ribeiro AL, Correa-Oliveira R, Teixeira MM. Chemokine receptor expression on the surface of peripheral blood mononuclear cells in Chagas disease. J Infect Dis. 15 janv 2004;189(2):214‑20. |
| 10.1086/381892 | Talvani A, Rocha MOC, Barcelos LS, Gomes YM, Ribeiro AL, Teixeira MM. Elevated concentrations of CCL2 and tumor necrosis factor-alpha in chagasic cardiomyopathy. Clin Infect Dis. 1 avr 2004;38(7):943‑50. |
| 10.1111/j.1399-0039.2004.00260.x | Moreno M, Silva EL, Ramírez LE, Palacio LG, Rivera D, Arcos-Burgos M. Chagas’ disease susceptibility/resistance: linkage disequilibrium analysis suggests epistasis between major histocompatibility complex and interleukin-10. Tissue Antigens. juill 2004;64(1):18‑24. |
| 10.1016/j.niox.2004.07.010 | Fabrino DL, Leon LL, Parreira GG, Genestra M, Almeida PE, Melo RCN. Peripheral blood monocytes show morphological pattern of activation and decreased nitric oxide production during acute Chagas’ disease in rats. Nitric Oxide. sept 2004;11(2):166‑74. |
| 10.1086/427515 | Machado FS, Koyama NS, Carregaro V, Ferreira BR, Milanezi CM, Teixeira MM, et al. CCR5 plays a critical role in the development of myocarditis and host protection in mice infected with *Trypanosoma cruzi*. J Infect Dis. 15 févr 2005;191(4):627‑36. |
| 10.1016/j.imlet.2004.10.017 | Rodríguez-Pérez JM, Cruz-Robles D, Hernández-Pacheco G, Pérez-Hernández N, Murguía LE, Granados J, et al. Tumor necrosis factor-alpha promoter polymorphism in Mexican patients with Chagas’ disease. Immunol Lett. 15 avr 2005;98(1):97‑102. |
| 10.1016/S0002-9440(10)62976-8 | Cunha-Neto E, Dzau VJ, Allen PD, Stamatiou D, Benvenutti L, Higuchi ML, et al. Cardiac Gene Expression Profiling Provides Evidence for Cytokinopathy as a Molecular Mechanism in Chagas’ Disease Cardiomyopathy. Am J Pathol. août 2005;167(2):305‑13. |
| 10.1128/IAI.73.12.7960-7966.2005 | Gomes JAS, Bahia-Oliveira LMG, Rocha MOC, Busek SCU, Teixeira MM, Silva JS, et al. Type 1 chemokine receptor expression in Chagas’ disease correlates with morbidity in cardiac patients. Infect Immun. déc 2005;73(12):7960‑6. |
| 10.1016/j.clim.2005.12.011 | Crema E, Monteiro I de O, Gomes MGZ, Silva AA, Rodrigues Júnior V. Evaluation of cytokines (MIG, IFN-gamma, TNF-alpha, IL-4, IL-5, and IL-10) during the different evolutive phases of chagasic esophagopathy. Clin Immunol. mai 2006;119(2):213‑8. |
| 10.1086/503368 | Ramasawmy R, Cunha-Neto E, Faé KC, Müller NG, Cavalcanti VL, Drigo SA, et al. BAT1, a putative anti-inflammatory gene, is associated with chronic Chagas cardiomyopathy. J Infect Dis. 15 mai 2006;193(10):1394‑9. |
| 10.1086/505395 | Ramasawmy R, Cunha-Neto E, Fae KC, Martello FG, Müller NG, Cavalcanti VL, et al. The monocyte chemoattractant protein-1 gene polymorphism is associated with cardiomyopathy in human chagas disease. Clin Infect Dis. 1 août 2006;43(3):305‑11. |
| 10.1016/j.freeradbiomed.2006.04.009 | Wen J jun, Yachelini PC, Sembaj A, Manzur RE, Garg NJ. Increased oxidative stress is correlated with mitochondrial dysfunction in chagasic patients. Free Radic Biol Med. 15 juill 2006;41(2):270‑6. |
| 10.1016/j.humimm.2006.06.004 | Flórez O, Zafra G, Morillo C, Martín J, González CI. Interleukin-1 gene cluster polymorphism in chagas disease in a Colombian case-control study. Hum Immunol. sept 2006;67(9):741‑8. |
| 10.1016/j.ijcard.2006.04.046 | de Oliveira TB, Pedrosa RC, Filho DW. Oxidative stress in chronic cardiopathy associated with Chagas disease. Int J Cardiol. 4 avr 2007;116(3):357‑63. |
| 10.1016/j.exppara.2006.12.007 | Ribeiro DA, Calvi SA, Picka MM, Persi E, de Carvalho TB, Caetano PK, et al. DNA damage and nitric oxide synthesis in experimentally infected Balb/c mice with *Trypanosoma cruzi*. Exp Parasitol. juill 2007;116(3):296‑301. |
| 10.1016/j.molimm.2007.04.015 | Ramasawmy R, Faé KC, Cunha-Neto E, Borba SCP, Ianni B, Mady C, et al. Variants in the promoter region of IKBL/NFKBIL1 gene may mark susceptibility to the development of chronic Chagas’ cardiomyopathy among *Trypanosoma cruzi*-infected individuals. Mol Immunol. janv 2008;45(1):283‑8. |
| 10.1111/j.1365-3083.2007.01987.x | Fonseca SG, Reis MM, Coelho V, Nogueira LG, Monteiro SM, Mairena EC, et al. Locally produced survival cytokines IL-15 and IL-7 may be associated to the predominance of CD8+ T cells at heart lesions of human chronic Chagas disease cardiomyopathy. Scand J Immunol. 2007;66(2‑3):362‑71. |
| 10.1086/523653 | Ramasawmy R, Fae KC, Cunha-Neto E, Müller NG, Cavalcanti VL, Ferreira RC, et al. Polymorphisms in the gene for lymphotoxin-alpha predispose to chronic Chagas cardiomyopathy. J Infect Dis. 15 déc 2007;196(12):1836‑43. |
| 10.1016/j.micinf.2007.04.010 | Zafra G, Morillo C, Martín J, González A, González CI. Polymorphism in the 3’ UTR of the IL12B gene is associated with Chagas’ disease cardiomyopathy. Microbes Infect. juill 2007;9(9):1049‑52. |
| 10.1086/587487 | Gutierrez FRS, Lalu MM, Mariano FS, Milanezi CM, Cena J, Gerlach RF, et al. Increased activities of cardiac matrix metalloproteinases matrix metalloproteinase (MMP)-2 and MMP-9 are associated with mortality during the acute phase of experimental *Trypanosoma cruzi* infection. J Infect Dis. 15 mai 2008;197(10):1468‑76. |
| 10.1086/529527 | Cuervo H, Pineda MA, Aoki MP, Gea S, Fresno M, Gironès N. Inducible nitric oxide synthase and arginase expression in heart tissue during acute *Trypanosoma cruzi* infection in mice: arginase I is expressed in infiltrating CD68+ macrophages. J Infect Dis. 15 juin 2008;197(12):1772‑82. |
| 10.1016/j.ijpara.2008.04.009 | Silvina Lo Presti M, Walter Rivarola H, Bustamante JM, Fernández AR, Enders JE, Levin G, et al. Some components of the cardiac β-adrenergic system are altered in the chronic indeterminate form of experimental *Trypanosoma cruzi* infection. Int J Parasitol. nov 2008;38(13):1481‑92. |
| 10.1016/j.ijmm.2008.08.005 | Calvet CM, Oliveira FOR, Araújo-Jorge TC, Pereira MCS. Regulation of extracellular matrix expression and distribution in *Trypanosoma cruzi*-infected cardiomyocytes. Int J Med Microbiol. avr 2009;299(4):301‑12. |
| 10.1086/596061 | Costa GC, da Costa Rocha MO, Moreira PR, Menezes CAS, Silva MR, Gollob KJ, et al. Functional IL-10 gene polymorphism is associated with Chagas disease cardiomyopathy. J Infect Dis. 1 févr 2009;199(3):451‑4. |
| 10.1016/j.cyto.2008.11.013 | Calzada JE, Beraún Y, González CI, Martín J. Transforming growth factor beta 1 (TGFbeta1) gene polymorphisms and Chagas disease susceptibility in Peruvian and Colombian patients. Cytokine. mars 2009;45(3):149‑53. |
| 10.1016/j.vetimm.2009.01.004 | Guedes PMM, Veloso VM, Afonso LCC, Caliari MV, Carneiro CM, Diniz LF, et al. Development of chronic cardiomyopathy in canine Chagas disease correlates with high IFN-gamma, TNF-alpha, and low IL-10 production during the acute infection phase. Vet Immunol Immunopathol. 15 juill 2009;130(1‑2):43‑52. |
| 10.1086/599212 | Ramasawmy R, Cunha-Neto E, Fae KC, Borba SCP, Teixeira PC, Ferreira SCP, et al. Heterozygosity for the S180L variant of MAL/TIRAP, a gene expressing an adaptor protein in the Toll-like receptor pathway, is associated with lower risk of developing chronic Chagas cardiomyopathy. J Infect Dis. 15 juin 2009;199(12):1838‑45. |
| 10.1186/1471-2164-10-252 | Costales JA, Daily JP, Burleigh BA. Cytokine-dependent and-independent gene expression changes and cell cycle block revealed in *Trypanosoma cruzi*-infected host cells by comparative mRNA profiling. BMC Genomics. 29 mai 2009;10:252. |
| 10.1093/eurjhf/hfp105 | Lula JF, Rocha MO da C, Nunes M do CP, Ribeiro ALP, Teixeira MM, Bahia MT, et al. Plasma concentrations of tumour necrosis factor-alpha, tumour necrosis factor-related apoptosis-inducing ligand, and FasLigand/CD95L in patients with Chagas cardiomyopathy correlate with left ventricular dysfunction. Eur J Heart Fail. sept 2009;11(9):825‑31. |
| 10.1016/j.micinf.2009.08.009 | Goldenberg RCDS, Iacobas DA, Iacobas S, Rocha LL, da Silva de Azevedo Fortes F, Vairo L, et al. Transcriptomic alterations in *Trypanosoma cruzi*-infected cardiac myocytes. Microbes Infect. déc 2009;11(14‑15):1140‑9. |
| 10.1080/08820130902729637 | Cruz-Robles D, Chávez-González JP, Cavazos-Quero MM, Pérez-Méndez O, Reyes PA, Vargas-Alarcón G. Association between IL-1B and IL-1RN gene polymorphisms and Chagas’ disease development susceptibility. Immunol Invest. 2009;38(3‑4):231‑9. |
| 10.1080/08820130902766589 | García Borrás S, Racca L, Cotorruelo C, Biondi C, Beloscar J, Racca A. Distribution of HLA-DRB1 alleles in Argentinean patients with Chagas’ disease cardiomyopathy. Immunol Invest. 2009;38(3‑4):268‑75. |
| 10.1590/s0074-02762009000500009 | Aguirre KLH, Alves JB, Silva GAB, Cardoso JE, Murta SMF, Ferreira AJ. Epidermal growth factor receptors, testosterone levels and parotid gland changes in rats infected with *Trypanosoma cruzi*. Mem Inst Oswaldo Cruz. août 2009;104(5):715‑9. |
| 10.1016/j.cyto.2009.09.012 | Ronco MT, Francés DE, Ingaramo PI, Quiroga AD, Alvarez ML, Pisani GB, et al. Tumor necrosis factor alpha induced by *Trypanosoma cruzi* infection mediates inflammation and cell death in the liver of infected mice. Cytokine. janv 2010;49(1):64‑72. |
| 10.1016/j.meegid.2010.03.009 | Torres OA, Calzada JE, Beraún Y, Morillo CA, González A, González CI, et al. Role of the IFNG +874T/A polymorphism in Chagas disease in a Colombian population. Infect Genet Evol. juill 2010;10(5):682‑5. |
| 10.4269/ajtmh.2010.09-0399 | Adesse D, Iacobas DA, Iacobas S, Garzoni LR, Meirelles M de N, Tanowitz HB, et al. Transcriptomic signatures of alterations in a myoblast cell line infected with four distinct strains of *Trypanosoma cruzi*. Am J Trop Med Hyg. mai 2010;82(5):846‑54. |
| 10.1016/j.jacc.2010.02.030 | Wen JJ, Gupta S, Guan Z, Dhiman M, Condon D, Lui C, et al. Phenyl-alpha-tert-butyl-nitrone and benzonidazole treatment controlled the mitochondrial oxidative stress and evolution of cardiomyopathy in chronic chagasic Rats. J Am Coll Cardiol. 1 juin 2010;55(22):2499‑508. |
| 10.1086/653481 | Soares MBP, de Lima RS, Rocha LL, Vasconcelos JF, Rogatto SR, dos Santos RR, et al. Gene expression changes associated with myocarditis and fibrosis in hearts of mice with chronic chagasic cardiomyopathy. J Infect Dis. 15 août 2010;202(3):416‑26. |
| 10.1016/j.vetimm.2010.06.010 | Guedes PMM, Veloso VM, Talvani A, Diniz LF, Caldas IS, Do-Valle-Matta MA, et al. Increased type 1 chemokine expression in experimental Chagas disease correlates with cardiac pathology in beagle dogs. Vet Immunol Immunopathol. 15 nov 2010;138(1‑2):106‑13. |
| 10.1111/j.1540-8159.2010.03025.x | Llaguno M, Pertili LAR de R, da Silva MV, Bunazar P, Reges AM, Faleiros ACG, et al. The relationship between heart rate variability and serum cytokines in chronic chagasic patients with persistent parasitemia. Pacing Clin Electrophysiol. juin 2011;34(6):724‑35. |
| 10.5858/135.2.243 | Saravia SGM, Haberland A, Bartel S, Araujo R, Valda G, Reynaga DD, et al. Cardiac troponin T measured with a highly sensitive assay for diagnosis and monitoring of heart injury in chronic Chagas disease. Arch Pathol Lab Med. févr 2011;135(2):243‑8. |
| 10.1016/j.jneuroim.2011.03.010 | Pérez AR, Silva-Barbosa SD, Berbert LR, Revelli S, Beloscar J, Savino W, et al. Immunoneuroendocrine alterations in patients with progressive forms of chronic Chagas disease. J Neuroimmunol. juin 2011;235(1‑2):84‑90. |
| 10.1371/journal.pntd.0001205 | Teixeira PC, Santos RHB, Fiorelli AI, Bilate AMB, Benvenuti LA, Stolf NA, et al. Selective decrease of components of the creatine kinase system and ATP synthase complex in chronic Chagas disease cardiomyopathy. PLoS Negl Trop Dis. juin 2011;5(6):e1205. |
| 10.1016/j.cyto.2011.12.007 | Criado L, Flórez O, Martín J, González CI. Genetic polymorphisms in TNFA/TNFR2 genes and Chagas disease in a Colombian endemic population. Cytokine. mars 2012;57(3):398‑401. |
| 10.1371/journal.pntd.0001587 | del Puerto F, Nishizawa JE, Kikuchi M, Roca Y, Avilas C, Gianella A, et al. Protective human leucocyte antigen haplotype, HLA-DRB1*01-B*14, against chronic Chagas disease in Bolivia. PLoS Negl Trop Dis. 2012;6(3):e1587. |
| 10.1074/mcp.M112.017640 | Wen JJ, Zago MP, Nuñez S, Gupta S, Burgos FN, Garg NJ. Serum Proteomic Signature of Human Chagasic Patients for the Identification of Novel Potential Protein Biomarkers of Disease *. Molecular & Cellular Proteomics. 1 août 2012;11(8):435‑52. |
| 10.1159/000337080 | Wang Y, Moreira M da CV, Khan A, Heringer-Walther S, Schultheiss HP, Wessel N, et al. Prognostic significance of circulating levels of hepatocyte growth factor in patients with chagas’ disease and idiopathic dilated cardiomyopathy. Cardiology. 2012;121(4):240‑6. |
| 10.1371/journal.pntd.0001644 | Carvalho CME, Silverio JC, da Silva AA, Pereira IR, Coelho JMC, Britto CC, et al. Inducible nitric oxide synthase in heart tissue and nitric oxide in serum of *Trypanosoma cruzi*-infected rhesus monkeys: association with heart injury. PLoS Negl Trop Dis. 2012;6(5):e1644. |
| 10.1016/j.parint.2012.07.008 | Souza V do CG, Schlemmer KB, Noal CB, Jaques JA dos S, Zimmermann CEP, Leal CAM, et al. E-NTPDase and E-ADA activities are altered in lymphocytes of patients with indeterminate form of Chagas’ disease. Parasitol Int. déc 2012;61(4):690‑6. |
| 10.1371/journal.pntd.0001867 | Nogueira LG, Santos RHB, Ianni BM, Fiorelli AI, Mairena EC, Benvenuti LA, et al. Myocardial chemokine expression and intensity of myocarditis in Chagas cardiomyopathy are controlled by polymorphisms in CXCL9 and CXCL10. PLoS Negl Trop Dis. 2012;6(10):e1867. |
| 10.1161/JAHA.112.003855 | Wan X, Gupta S, Zago MP, Davidson MM, Dousset P, Amoroso A, et al. Defects of mtDNA replication impaired mitochondrial biogenesis during *Trypanosoma cruzi* infection in human cardiomyocytes and chagasic patients: the role of Nrf1/2 and antioxidant response. J Am Heart Assoc. déc 2012;1(6):e003855. |
| 10.1371/journal.pntd.0002034 | Corral RS, Guerrero NA, Cuervo H, Gironès N, Fresno M. *Trypanosoma cruzi* infection and endothelin-1 cooperatively activate pathogenic inflammatory pathways in cardiomyocytes. PLoS Negl Trop Dis. 2013;7(2):e2034. |
| 10.1016/j.cyto.2012.12.018 | Wang Y, Khan A, Heringer-Walther S, Schultheiss HP, Moreira M da CV, Walther T. Prognostic value of circulating levels of stem cell growth factor beta (SCGF beta) in patients with Chagas’ disease and idiopathic dilated cardiomyopathy. Cytokine. mars 2013;61(3):728‑31. |
| 10.1016/j.humimm.2013.01.023 | del Puerto F, Kikuchi M, Nishizawa JE, Roca Y, Avila C, Gianella A, et al. 21-Hydroxylase gene mutant allele CYP21A2*15 strongly linked to the resistant HLA haplotype B*14:02-DRB1*01:02 in chronic Chagas disease. Hum Immunol. juin 2013;74(6):783‑6. |
| 10.1371/journal.pone.0057181 | Cutrullis RA, Petray PB, Schapachnik E, Sánchez R, Postan M, González MN, et al. Elevated serum levels of macrophage migration inhibitory factor are associated with progressive chronic cardiomyopathy in patients with Chagas disease. PLoS One. 2013;8(2):e57181. |
| 10.1016/j.ahj.2013.01.001 | Bautista-López NL, Morillo CA, López-Jaramillo P, Quiroz R, Luengas C, Silva SY, et al. Matrix metalloproteinases 2 and 9 as diagnostic markers in the progression to Chagas cardiomyopathy. Am Heart J. avr 2013;165(4):558‑66. |
| 10.1371/journal.pone.0060237 | Luz PR, Boldt ABW, Grisbach C, Kun JFJ, Velavan TP, Messias-Reason IJT. Association of L-Ficolin Levels and FCN2 Genotypes with Chronic Chagas Disease. PLOS ONE. 4 avr 2013;8(4):e60237. |
| 10.1128/IAI.00153-13 | Fares RCG, Gomes J de AS, Garzoni LR, Waghabi MC, Saraiva RM, Medeiros NI, et al. Matrix metalloproteinases 2 and 9 are differentially expressed in patients with indeterminate and cardiac clinical forms of Chagas disease. Infect Immun. oct 2013;81(10):3600‑8. |
| 10.1161/JAHA.113.000302 | Dhiman M, Wan X, Popov VL, Vargas G, Garg NJ. MnSODtg mice control myocardial inflammatory and oxidative stress and remodeling responses elicited in chronic Chagas disease. J Am Heart Assoc. 17 oct 2013;2(5):e000302. |
| 10.1016/j.ijcard.2013.08.110 | Luz PR, Velavan TP, Kremsner PG, Messias-Reason IJT. Association of IP-10 and PDGF-BB levels with clinical forms of chronic Chagas disease. Int J Cardiol. 15 nov 2013;169(4):e53-55. |
| 10.1371/journal.pone.0078367 | Dias FC, Medina T da S, Mendes-Junior CT, Dantas RO, Pissetti CW, Rodrigues Junior V, et al. Polymorphic sites at the immunoregulatory CTLA-4 gene are associated with chronic chagas disease and its clinical manifestations. PLoS One. 2013;8(10):e78367. |
| 10.1371/journal.pone.0079629 | Deng X, Sabino EC, Cunha-Neto E, Ribeiro AL, Ianni B, Mady C, et al. Genome wide association study (GWAS) of Chagas cardiomyopathy in *Trypanosoma cruzi* seropositive subjects. PLoS One. 2013;8(11):e79629. |
| 10.1186/1471-2334-13-587 | Frade AF, Pissetti CW, Ianni BM, Saba B, Lin-Wang HT, Nogueira LG, et al. Genetic susceptibility to Chagas disease cardiomyopathy: involvement of several genes of the innate immunity and chemokine-dependent migration pathways. BMC Infect Dis. 12 déc 2013;13:587. |
| 10.1371/journal.pone.0083446 | Frade AF, Teixeira PC, Ianni BM, Pissetti CW, Saba B, Wang LHT, et al. Polymorphism in the Alpha Cardiac Muscle Actin 1 Gene Is Associated to Susceptibility to Chronic Inflammatory Cardiomyopathy. PLoS One [Internet]. 19 déc 2013 [cité 10 août 2020];8(12). Disponible sur: https://www.ncbi.nlm.nih.gov/pmc/articles/PMC3868584/ |
| 10.1016/j.molimm.2013.11.007 | de Souza SM, Vieira PM de A, Roatt BM, Reis LES, da Silva Fonseca K, Nogueira NC, et al. Dogs infected with the blood trypomastigote form of *Trypanosoma cruzi* display an increase expression of cytokines and chemokines plus an intense cardiac parasitism during acute infection. Mol Immunol. mars 2014;58(1):92‑7. |
| 10.1371/journal.pone.0087082 | Sousa GR, Gomes JAS, Fares RCG, Damásio MP de S, Chaves AT, Ferreira KS, et al. Plasma cytokine expression is associated with cardiac morbidity in chagas disease. PLoS One. 2014;9(3):e87082. |
| 10.1016/j.ijcard.2014.05.019 | Ferreira LRP, Frade AF, Santos RHB, Teixeira PC, Baron MA, Navarro IC, et al. MicroRNAs miR-1, miR-133a, miR-133b, miR-208a and miR-208b are dysregulated in Chronic Chagas disease Cardiomyopathy. Int J Cardiol. 20 août 2014;175(3):409‑17. |
| 10.1590/0074-0276140033 | Pereira IR, Vilar-Pereira G, Silva AA da, Lannes-Vieira J. Severity of chronic experimental Chagas’ heart disease parallels tumour necrosis factor and nitric oxide levels in the serum: models of mild and severe disease. Mem Inst Oswaldo Cruz. juin 2014;109(3):289‑98. |
| 10.1155/2014/914326 | Nogueira LG, Santos RHB, Fiorelli AI, Mairena EC, Benvenuti LA, Bocchi EA, et al. Myocardial gene expression of T-bet, GATA-3, Ror-γt, FoxP3, and hallmark cytokines in chronic Chagas disease cardiomyopathy: an essentially unopposed TH1-type response. Mediators Inflamm. 2014;2014:914326. |
| 10.1371/journal.pntd.0003227 | Okamoto EE, Sherbuk JE, Clark EH, Marks MA, Gandarilla O, Galdos-Cardenas G, et al. Biomarkers in *Trypanosoma cruzi*-infected and uninfected individuals with varying severity of cardiomyopathy in Santa Cruz, Bolivia. PLoS Negl Trop Dis. oct 2014;8(10):e3227. |
| 10.1016/j.humimm.2014.09.023 | Machuca MA, Suárez EU, Echeverría LE, Martín J, González CI. SNP/haplotype associations of CCR2 and CCR5 genes with severity of chagasic cardiomyopathy. Hum Immunol. déc 2014;75(12):1210‑5. |
| 10.1016/j.bbi.2014.11.016 | 1González FB, Villar SR, Fernández Bussy R, Martin GH, Pérol L, Manarin R, et al. Immunoendocrine dysbalance during uncontrolled *T. cruzi* infection is associated with the acquisition of a Th-1-like phenotype by Foxp3(+) T cells. Brain Behav Immun. mars 2015;45:219‑32. |
| 10.1155/2015/595829 | Dias FC, Mendes-Junior CT, Silva MC, Tristão FSM, Dellalibera-Joviliano R, Moreau P, et al. Human leucocyte antigen-G (HLA-G) and its murine functional homolog Qa2 in the *Trypanosoma cruzi* Infection. Mediators Inflamm. 2015;2015:595829. |
| 10.1111/pim.12183 | Vasconcelos RHT, Azevedo E de AN, Diniz GTN, Cavalcanti M da GA de M, de Oliveira W, de Morais CNL, et al. Interleukin-10 and tumour necrosis factor-alpha serum levels in chronic Chagas disease patients. Parasite Immunol. juill 2015;37(7):376‑9. |
| 10.1016/j.cyto.2015.01.037 | Nogueira LG, Frade AF, Ianni BM, Laugier L, Pissetti CW, Cabantous S, et al. Functional IL18 polymorphism and susceptibility to Chronic Chagas Disease. Cytokine. mai 2015;73(1):79‑83. |
| 10.1016/j.yexmp.2015.03.034 | Báez AL, Reynoso MN, Lo Presti MS, Bazán PC, Strauss M, Miler N, et al. Mitochondrial dysfunction in skeletal muscle during experimental Chagas disease. Exp Mol Pathol. juin 2015;98(3):467‑75. |
| 10.1016/j.ijcard.2015.04.106 | Lidani KCF, Beltrame MH, Luz PR, Sandri TL, Nisihara RM, de Messias-Reaso IJ. Is pentraxin 3 a cardiovascular marker in patients with chronic Chagas disease? Int J Cardiol. 2015;190:233‑5. |
| 10.1371/journal.pntd.0003828 | Navarro IC, Ferreira FM, Nakaya HI, Baron MA, Vilar-Pereira G, Pereira IR, et al. MicroRNA Transcriptome Profiling in Heart of *Trypanosoma cruzi*-Infected Mice: Parasitological and Cardiological Outcomes. PLoS Negl Trop Dis [Internet]. 18 juin 2015 [cité 15 avr 2021];9(6). Disponible sur: https://www.ncbi.nlm.nih.gov/pmc/articles/PMC4473529/ |
| 10.1371/journal.pone.0131447 | Barbosa-Ferreira JM, Mady C, Ianni BM, Lopes HF, Ramires FJA, Salemi VMC, et al. Dysregulation of Autonomic Nervous System in Chagas’ Heart Disease Is Associated with Altered Adipocytokines Levels. PLoS One. 2015;10(7):e0131447. |
| 10.1016/j.ijcard.2015.07.040 | Keating SM, Deng X, Fernandes F, Cunha-Neto E, Ribeiro AL, Adesina B, et al. Inflammatory and cardiac biomarkers are differentially expressed in clinical stages of Chagas disease. Int J Cardiol. 15 nov 2015;199:451‑9. |
| 10.3389/fimmu.2015.00428 | Linhares-Lacerda L, Palu CC, Ribeiro-Alves M, Paredes BD, Morrot A, Garcia-Silva MR, et al. Differential Expression of microRNAs in Thymic Epithelial Cells from *Trypanosoma cruzi* Acutely Infected Mice: Putative Role in Thymic Atrophy. Front Immunol. 2015;6:428. |
| 10.1038/gene.2015.42 | Leon Rodriguez DA, Echeverría LE, González CI, Martin J. Investigation of the role of IL17A gene variants in Chagas disease. Genes Immun. déc 2015;16(8):536‑40. |
| 10.1371/journal.pone.0141847 | Oliveira AP de, Bernardo CR, Camargo AV da S, Ronchi LS, Borim AA, Mattos CCB de, et al. Genetic Susceptibility to Cardiac and Digestive Clinical Forms of Chronic Chagas Disease: Involvement of the CCR5 59029 A/G Polymorphism. PLOS ONE. 23 nov 2015;10(11):e0141847. |
| 10.1093/infdis/jiv561 | Juiz NA, Cayo NM, Burgos M, Salvo ME, Nasser JR, Búa J, et al. Human Polymorphisms in Placentally Expressed Genes and Their Association With Susceptibility to Congenital *Trypanosoma cruzi* Infection. J Infect Dis. 15 avr 2016;213(8):1299‑306. |
| 10.1371/journal.pntd.0004269 | Pinazo MJ, Posada E de J, Izquierdo L, Tassies D, Marques AF, de Lazzari E, et al. Altered Hypercoagulability Factors in Patients with Chronic Chagas Disease: Potential Biomarkers of Therapeutic Response. PLoS Negl Trop Dis. janv 2016;10(1):e0004269. |
| 10.1371/journal.pntd.0004257 | Luz PR, Miyazaki MI, Chiminacio Neto N, Padeski MC, Barros ACM, Boldt ABW, et al. Genetically Determined MBL Deficiency Is Associated with Protection against Chronic Cardiomyopathy in Chagas Disease. Tanowitz HB, éditeur. PLoS Negl Trop Dis. 8 janv 2016;10(1):e0004257. |
| 10.1371/journal.pntd.0003747 | Udoko AN, Johnson CA, Dykan A, Rachakonda G, Villalta F, Mandape SN, et al. Early Regulation of Profibrotic Genes in Primary Human Cardiac Myocytes by *Trypanosoma cruz*i. Burke M, éditeur. PLoS Negl Trop Dis. 15 janv 2016;10(1):e0003747. |
| 10.1016/j.actatropica.2016.01.025 | Rodrigues AA, Notário AFO, Teixeira TL, e Silva RT, Quintal APN, Alves RN, et al. A high throughput analysis of cytokines and chemokines expression during the course of *Trypanosoma cruzi* experimental oral infection. Acta Trop. mai 2016;157:42‑53. |
| 10.1093/infdis/jiw095 | Frade AF, Laugier L, Ferreira LRP, Baron MA, Benvenuti LA, Teixeira PC, et al. Myocardial Infarction-Associated Transcript, a Long Noncoding RNA, Is Overexpressed During Dilated Cardiomyopathy Due to Chronic Chagas Disease. J Infect Dis. 01 2016;214(1):161‑5. |
| 10.1371/journal.pntd.0004583 | Leon Rodriguez DA, Carmona FD, Echeverría LE, González CI, Martin J. IL18 Gene Variants Influence the Susceptibility to Chagas Disease. Hirayama K, éditeur. PLoS Negl Trop Dis. 30 mars 2016;10(3):e0004583. |
| 10.1038/srep31263 | Leon Rodriguez DA, Carmona FD, González CI, Martin J. Evaluation of VDR gene polymorphisms in *Trypanosoma cruzi* infection and chronic Chagasic cardiomyopathy. Sci Rep. nov 2016;6(1):31263. |
| 10.1371/journal.pone.0159197 | Houston-Ludlam GA, Belew AT, El-Sayed NM. Comparative Transcriptome Profiling of Human Foreskin Fibroblasts Infected with the Sylvio and Y Strains of *Trypanosoma cruzi*. Solari A, éditeur. PLoS ONE. 9 août 2016;11(8):e0159197. |
| 10.1016/j.ijcard.2016.10.098 | Echeverría LE, Rojas LZ, Calvo LS, Roa ZM, Rueda-Ochoa OL, Morillo CA, et al. Profiles of cardiovascular biomarkers according to severity stages of Chagas cardiomyopathy. International Journal of Cardiology. janv 2017;227:577‑82. |
| 10.1016/j.cyto.2016.12.002 | de Oliveira AP, Ayo CM, Mimura KKO, Oliani SM, Bernardo CR, Camargo AVS, et al. Plasma concentrations of CCL3 and CCL4 in the cardiac and digestive clinical forms of chronic Chagas disease. Cytokine. mars 2017;91:51‑6. |
| 10.1093/infdis/jiw540 | Ferreira LRP, Ferreira FM, Nakaya HI, Deng X, Cândido D da S, de Oliveira LC, et al. Blood Gene Signatures of Chagas Cardiomyopathy With or Without Ventricular Dysfunction. J Infect Dis. 1 févr 2017;215(3):387‑95. |
| 10.1016/j.micpath.2017.01.002 | Souza V do CG, dos Santos JT, Cabral FL, Barbisan F, Azevedo MI, Dias Carli LF, et al. Evaluation of P2X7 receptor expression in peripheral lymphocytes and immune profile from patients with indeterminate form of Chagas disease. Microbial Pathogenesis. mars 2017;104:32‑8. |
| 10.1371/journal.pntd.0005436 | Juiz NA, Solana ME, Acevedo GR, Benatar AF, Ramirez JC, da Costa PA, et al. Different genotypes of *Trypanosoma cruzi* produce distinctive placental environment genetic response in chronic experimental infection. Lv S, éditeur. PLoS Negl Trop Dis. 8 mars 2017;11(3):e0005436. |
| 10.1371/journal.pone.0172833 | Sousa GR, Gomes JAS, Damasio MPS, Nunes MCP, Costa HS, Medeiros NI, et al. The role of interleukin 17-mediated immune response in Chagas disease: High level is correlated with better left ventricular function. Eugenin EA, éditeur. PLoS ONE. 9 mars 2017;12(3):e0172833. |
| 10.1016/j.ajpath.2017.01.016 | Souza BS de F, Silva DN, Carvalho RH, Sampaio GL de A, Paredes BD, Aragão França L, et al. Association of Cardiac Galectin-3 Expression, Myocarditis, and Fibrosis in Chronic Chagas Disease Cardiomyopathy. The American Journal of Pathology. mai 2017;187(5):1134‑46. |
| 10.1155/2017/1017621 | Reis PG, Ayo CM, de Mattos LC, Brandão de Mattos C de C, Sakita KM, de Moraes AG, et al. Genetic Polymorphisms of IL17 and Chagas Disease in the South and Southeast of Brazil. J Immunol Res. 2017;2017:1017621. |
| 10.1016/j.prp.2017.04.014 | Beghini M, de Araújo MF, Severino VO, Etchebehere RM, Rocha Rodrigues DB, de Lima Pereira SA. Evaluation of the immunohistochemical expression of Gal-1, Gal-3 and Gal-9 in the colon of chronic chagasic patients. Pathology - Research and Practice. sept 2017;213(9):1207‑14. |
| 10.1093/cid/cix506 | Laugier L, Frade AF, Ferreira FM, Baron MA, Teixeira PC, Cabantous S, et al. Whole-Genome Cardiac DNA Methylation Fingerprint and Gene Expression Analysis Provide New Insights in the Pathogenesis of Chronic Chagas Disease Cardiomyopathy. Clin Infect Dis. 01 2017;65(7):1103‑11. |
| 10.1590/0037-8682-0015-2017 | Fu KYJ, Zamudio R, Henderson-Frost J, Almuedo A, Steinberg H, Clipman SJ, et al. Association of caspase-1 polymorphisms with Chagas cardiomyopathy among individuals in Santa Cruz, Bolivia. Rev Soc Bras Med Trop. août 2017;50(4):516‑23. |
| 10.1590/0037-8682-0025-2017 | Miranda MB de, Melo AS de, Almeida MS, Marinho SM, Oliveira Junior W, Gomes Y de M. Ex vivo T-lymphocyte chemokine receptor phenotypes in patients with chronic Chagas disease. Rev Soc Bras Med Trop. sept 2017;50(5):689‑92. |
| 10.1038/s41598-017-18080-9 | Ferreira LRP, Ferreira FM, Laugier L, Cabantous S, Navarro IC, da Silva Cândido D, et al. Integration of miRNA and gene expression profiles suggest a role for miRNAs in the pathobiological processes of acute *Trypanosoma cruzi* infection. Sci Rep. 21 déc 2017;7(1):17990. |
| 10.1016/j.actatropica.2017.11.009 | De Alba-Alvarado M, Salazar-Schettino PM, Jiménez-Álvarez L, Cabrera-Bravo M, García-Sancho C, Zenteno E, et al. Th-17 cytokines are associated with severity of *Trypanosoma cruzi* chronic infection in pediatric patients from endemic areas of Mexico. Acta Tropica. févr 2018;178:134‑41. |
| 10.1038/s41598-017-18937-z | Sandri TL, Lidani KCF, Andrade FA, Meyer CG, Kremsner PG, de Messias-Reason IJ, et al. Human complement receptor type 1 (CR1) protein levels and genetic variants in chronic Chagas Disease. Sci Rep. déc 2018;8(1):526. |
| 10.1371/journal.pone.0192378 | Clipman SJ, Henderson-Frost J, Fu KY, Bern C, Flores J, Gilman RH. Genetic association study of NLRP1, CARD, and CASP1 inflammasome genes with chronic Chagas cardiomyopathy among *Trypanosoma cruzi* seropositive patients in Bolivia. Ojcius DM, éditeur. PLoS ONE. 13 févr 2018;13(2):e0192378. |
| 10.1590/0074-02760170440 | Curvo EO, Ferreira RR, Madeira FS, Alves GF, Chambela MC, Mendes VG, et al. Correlation of transforming growth factor-β1 and tumour necrosis factor levels with left ventricular function in Chagas disease. Mem Inst Oswaldo Cruz [Internet]. 19 févr 2018 [cité 14 déc 2022];113(4). Disponible sur: http://www.scielo.br/scielo.php?script=sci_arttext&pid=S0074-02762018000400305&lng=en&tlng=en |
| 10.1016/j.ajpath.2018.02.011 | Juiz NA, Torrejón I, Burgos M, Torres AMF, Duffy T, Cayo NM, et al. Alterations in Placental Gene Expression of Pregnant Women with Chronic Chagas Disease. The American Journal of Pathology. juin 2018;188(6):1345‑53. |
| 10.3389/fmicb.2018.00269 | Linhares-Lacerda L, Granato A, Gomes-Neto JF, Conde L, Freire-de-Lima L, de Freitas EO, et al. Circulating Plasma MicroRNA-208a as Potential Biomarker of Chronic Indeterminate Phase of Chagas Disease. Front Microbiol. 6 mars 2018;9:269. |
| 10.3389/fimmu.2018.00615 | Batista AM, Alvarado-Arnez LE, Alves SM, Melo G, Pereira IR, Ruivo LA de S, et al. Genetic Polymorphism at CCL5 Is Associated With Protection in Chagas’ Heart Disease: Antagonistic Participation of CCR1+ and CCR5+ Cells in Chronic Chagasic Cardiomyopathy. Front Immunol. 11 avr 2018;9:615. |
| 10.1590/0074-02760170489 | Alvarado-Arnez LE, Batista AM, Alves SM, Melo G, Lorena VMB de, Cardoso CC, et al. Single nucleotide polymorphisms of cytokine-related genes and association with clinical outcome in a Chagas disease case-control study from Brazil. Mem Inst Oswaldo Cruz [Internet]. 14 mai 2018 [cité 14 déc 2022];113(6). Disponible sur: http://www.scielo.br/scielo.php?script=sci_arttext&pid=S0074-02762018000600301&lng=en&tlng=en |
| 10.1371/journal.pntd.0006589 | Pereira N de S, Queiroga TBD, Nunes DF, Andrade C de M, Nascimento MSL, Do-Valle-Matta MA, et al. Innate immune receptors over expression correlate with chronic chagasic cardiomyopathy and digestive damage in patients. Tanowitz HB, éditeur. PLoS Negl Trop Dis. 25 juill 2018;12(7):e0006589. |
| 10.1159/000491699 | González F, Villar S, D’Attilio L, Leiva R, Marquez J, Lioi S, et al. Dysregulated Network of Immune, Endocrine and Metabolic Markers is Associated to More Severe Human Chronic Chagas Cardiomyopathy. Neuroimmunomodulation. 2018;25(3):119‑28. |
| 10.1371/journal.pntd.0007324 | Sandri TL, Andrade FA, Lidani KCF, Einig E, Boldt ABW, Mordmüller B, et al. Human collectin-11 (COLEC11) and its synergic genetic interaction with MASP2 are associated with the pathophysiology of Chagas Disease. Santiago H da C, éditeur. PLoS Negl Trop Dis. 17 avr 2019;13(4):e0007324. |
| 10.3389/fimmu.2019.01671 | Gómez-Olarte S, Bolaños NI, Echeverry M, Rodríguez AN, Cuéllar A, Puerta CJ, et al. Intermediate Monocytes and Cytokine Production Associated With Severe Forms of Chagas Disease. Front Immunol. 19 juill 2019;10:1671. |
| 10.3390/ijms20164064 | Nonaka CKV, Macêdo CT, Cavalcante BRR, Alcântara AC de, Silva DN, Bezerra M da R, et al. Circulating miRNAs as Potential Biomarkers Associated with Cardiac Remodeling and Fibrosis in Chagas Disease Cardiomyopathy. Int J Mol Sci. 20 août 2019;20(16):E4064. |
| 10.1590/0037-8682-0133-2019 | Oliveira Junior LR de, Carvalho TB, Santos RM dos, Costa ÉAPN da, Pereira PCM, Kurokawa CS. Association of vitamin D3, VDR gene polymorphisms, and LL-37 with a clinical form of Chagas Disease. Rev Soc Bras Med Trop. 2019;52:e20190133. |
| 10.1038/s41598-019-50791-z | Medeiros NI, Gomes JAS, Fiuza JA, Sousa GR, Almeida EF, Novaes RO, et al. MMP-2 and MMP-9 plasma levels are potential biomarkers for indeterminate and cardiac clinical forms progression in chronic Chagas disease. Sci Rep. déc 2019;9(1):14170. |
| 10.1371/journal.pntd.0007859 | Strauss M, Acosta-Herrera M, Alcaraz A, Casares-Marfil D, Bosch-Nicolau P, Lo Presti MS, et al. Association of IL18 genetic polymorphisms with Chagas disease in Latin American populations. PLoS Negl Trop Dis [Internet]. 21 nov 2019 [cité 10 août 2020];13(11). Disponible sur: https://www.ncbi.nlm.nih.gov/pmc/articles/PMC6894881/ |
| 10.1371/journal.pone.0225588 | Ty MC, Loke P, Alberola J, Rodriguez A, Rodriguez-Cortes A. Immuno-metabolic profile of human macrophages after Leishmania and *Trypanosoma cruzi* infection. Kelly BL, éditeur. PLoS ONE. 16 déc 2019;14(12):e0225588. |
| 10.4269/ajtmh.19-0550 | Salvador F, Sánchez-Montalvá A, Martínez-Gallo M, Sulleiro E, Franco-Jarava C, Sao Avilés A, et al. Serum IL-10 Levels and Its Relationship with Parasitemia in Chronic Chagas Disease Patients. The American Journal of Tropical Medicine and Hygiene. 8 janv 2020;102(1):159‑63. |
| 10.1038/s41598-020-61965-5 | Strauss M, Palma-Vega M, Casares-Marfil D, Bosch-Nicolau P, Lo Presti MS, Molina I, et al. Genetic polymorphisms of IL17A associated with Chagas disease: results from a meta-analysis in Latin American populations. Sci Rep. 19 mars 2020;10(1):5015. |
| 10.1371/journal.pntd.0008162 | Mijares A, Espinosa R, Adams J, Lopez JR. Increases in [IP3]i aggravates diastolic [Ca2+] and contractile dysfunction in Chagas’ human cardiomyocytes. Santiago H da C, éditeur. PLoS Negl Trop Dis. 10 avr 2020;14(4):e0008162. |
| 10.1186/s40249-020-00663-w | de Araújo FF, Lima Torres KC, Viana Peixoto S, Pinho Ribeiro AL, Vaz Melo Mambrini J, Bortolo Rezende V, et al. CXCL9 and CXCL10 display an age-dependent profile in Chagas patients: a cohort study of aging in Bambui, Brazil. Infect Dis Poverty. déc 2020;9(1):51. |
| 10.3389/fcimb.2020.00255 | Nisimura LM, Coelho LL, de Melo TG, Vieira P de C, Victorino PH, Garzoni LR, et al. *Trypanosoma cruzi* Promotes Transcriptomic Remodeling of the JAK/STAT Signaling and Cell Cycle Pathways in Myoblasts. Front Cell Infect Microbiol. 17 juin 2020;10:255. |
| 10.3389/fimmu.2020.01386 | Frade-Barros AF, Ianni BM, Cabantous S, Pissetti CW, Saba B, Lin-Wang HT, et al. Polymorphisms in Genes Affecting Interferon-γ Production and Th1 T Cell Differentiation Are Associated With Progression to Chagas Disease Cardiomyopathy. Front Immunol [Internet]. 7 juill 2020 [cité 10 août 2020];11. Disponible sur: https://www.ncbi.nlm.nih.gov/pmc/articles/PMC7358543/ |
| 10.1371/journal.ppat.1008781 | Oliveira AER, Pereira MCA, Belew AT, Ferreira LRP, Pereira LMN, Neves EGA, et al. Gene expression network analyses during infection with virulent and avirulent *Trypanosoma cruzi* strains unveil a role for fibroblasts in neutrophil recruitment and activation. Hill KL, éditeur. PLoS Pathog. 18 août 2020;16(8):e1008781. |
| 10.1371/journal.pntd.0008667 | Pereira N de S, Queiroga TBD, da Silva DD, Nascimento MSL, Andrade CM de, Souto JT de, et al. NOD2 receptor is crucial for protecting against the digestive form of Chagas disease. Almeida IC, éditeur. PLoS Negl Trop Dis. 28 sept 2020;14(9):e0008667. |
| 10.1038/s41598-020-74540-9 | Jung H, Han S, Lee Y. Transcriptome analysis of alternative splicing in the pathogen life cycle in human foreskin fibroblasts infected with *Trypanosoma cruzi*. Sci Rep. 15 oct 2020;10(1):17481. |
| 10.3389/fimmu.2020.572178 | Rada J, Donato M, Penas FN, Alba Soto C, Cevey ÁC, Pieralisi AV, et al. IL-10-Dependent and -Independent Mechanisms Are Involved in the Cardiac Pathology Modulation Mediated by Fenofibrate in an Experimental Model of Chagas Heart Disease. Front Immunol. 24 sept 2020;11:572178. |
| 10.3389/fgene.2020.01031 | Castro TBR de, Canesso MCC, Boroni M, Chame DF, Souza D de L, Toledo NE de, et al. Differential Modulation of Mouse Heart Gene Expression by Infection With Two *Trypanosoma cruzi* Strains: A Transcriptome Analysis. Frontiers in Genetics [Internet]. 2020 [cité 22 juill 2022];11. Disponible sur: https://www.frontiersin.org/articles/10.3389/fgene.2020.01031 |
| 10.1590/0037-8682-0566-2019 | Lassen O, Tabares S, Bertolotto P, Ojeda S, Sembaj A. Preliminary study between Y chromosome haplogroups and chagasic cardiomyopathy manifestations in patients with Chagas disease. Rev Soc Bras Med Trop. 2020;53:e20190566. |
| 10.3389/fimmu.2020.595250 | Medina L, Castillo C, Liempi A, Guerrero-Muñoz J, Rojas-Pirela M, Maya JD, et al. *Trypanosoma cruzi* and Toxoplasma gondii Induce a Differential MicroRNA Profile in Human Placental Explants. Front Immunol. 6 nov 2020;11:595250. |
| 10.1371/journal.pntd.0008889 | Laugier L, Ferreira LRP, Ferreira FM, Cabantous S, Frade AF, Nunes JP, et al. miRNAs may play a major role in the control of gene expression in key pathobiological processes in Chagas disease cardiomyopathy. PLOS Neglected Tropical Diseases. 22 déc 2020;14(12):e0008889. |
| 10.1016/j.parint.2020.102213 | Blasco RL, Strauss M, Velázquez López DA, Tabares S, Sembaj A, Rivarola HW, et al. SCN5A gene variants as potential markers of the progression of chronic chagasic cardiac alterations. Parasitology International. févr 2021;80:102213. |
| 10.1016/j.meegid.2020.104671 | Silva MC, da Silva Medina T, Fuzo CA, Dias FC, Freitas-Castro F, Fukutani KF, et al. Polymorphism in the catalytic subunit of the PI3Kγ gene is associated with *Trypanosoma cruzi*-induced chronic chagasic cardiomyopathy. Infection, Genetics and Evolution. mars 2021;88:104671. |
| 10.1093/cid/ciab090 | Casares-Marfil D, Strauss M, Bosch-Nicolau P, Lo Presti MS, Molina I, Chevillard C, et al. A Genome-Wide Association Study Identifies Novel Susceptibility loci in Chronic Chagas Cardiomyopathy. Clin Infect Dis. 16 août 2021;73(4):672‑9. |
| 10.1371/journal.pone.0246692 | do Carmo Neto JR, Vinicius da Silva M, Braga YLL, Florencio da Costa AW, Fonseca SG, Nagib PRA, et al. Correlation between intestinal BMP2, IFNγ, and neural death in experimental infection with *Trypanosoma cruzi*. Melo RCN, éditeur. PLoS ONE. 9 févr 2021;16(2):e0246692. |
| 10.3389/fimmu.2020.539086 | Borghi SM, Fattori V, Carvalho TT, Tatakihara VLH, Zaninelli TH, Pinho-Ribeiro FA, et al. Experimental *Trypanosoma cruzi* Infection Induces Pain in Mice Dependent on Early Spinal Cord Glial Cells and NFκB Activation and Cytokine Production. Front Immunol. 26 janv 2021;11:539086. |
| 10.36660/abc.20190403 | Fernandes F, Moreira CHV, Oliveira LC, Souza-Basqueira M, Ianni BM, Lorenzo C di, et al. Galectin-3 Associated with Severe Forms and Long-term Mortality in Patients with Chagas Disease. Arq Bras Cardiol. févr 2021;116(2):248‑56. |
| 10.1111/pim.12829 | Lidani KCF, Andrade FA, Beltrame MH, Chakravarti I, Tizzot MR, Cavalcanti EO, et al. Ficolin‐3 in chronic Chagas disease: Low serum levels associated with the risk of cardiac insufficiency. Parasite Immunol [Internet]. juin 2021 [cité 14 déc 2022];43(6). Disponible sur: https://onlinelibrary.wiley.com/doi/10.1111/pim.12829 |
| 10.1371/journal.ppat.1009502 | Florentino PTV, Mendes D, Vitorino FNL, Martins DJ, Cunha JPC, Mortara RA, et al. DNA damage and oxidative stress in human cells infected by *Trypanosoma cruzi*. McCulloch R, éditeur. PLoS Pathog. 7 avr 2021;17(4):e1009502. |
| 10.1016/j.biochi.2021.04.005 | Musikant D, Higa R, Rodríguez CE, Edreira MM, Campetella O, Jawerbaum A, et al. Sialic acid removal by trans-sialidase modulates MMP-2 activity during *Trypanosoma cruzi* infection. Biochimie. juill 2021;186:82‑93. |
| 10.1186/s40659-021-00345-3 | Ballinas-Verdugo MA, Jiménez-Ortega RF, Martínez-Martínez E, Rivas N, Contreras-López EA, Carbó R, et al. Circulating miR-146a as a possible candidate biomarker in the indeterminate phase of Chagas disease. Biol Res. 21 juill 2021;54(1):21. |
| 10.1128/Spectrum.00364-21 | Choudhuri S, Bhavnani SK, Zhang W, Botelli V, Barrientos N, Iñiguez F, et al. Prognostic Performance of Peripheral Blood Biomarkers in Identifying Seropositive Individuals at Risk of Developing Clinically Symptomatic Chagas Cardiomyopathy. Martin RM, éditeur. Microbiol Spectr. 3 sept 2021;9(1):e00364-21. |
| 10.1016/j.meegid.2021.105079 | Zhou L, Li Z, Li J, Yang S, Gong H. Detecting imperative genes and infiltrating immune cells in chronic Chagas cardiomyopathy by bioinformatics analysis. Infection, Genetics and Evolution. nov 2021;95:105079. |
| 10.3389/fcimb.2021.722984 | Gómez I, Thomas MC, Palacios G, Egui A, Carrilero B, Simón M, et al. Differential Expression of Immune Response Genes in Asymptomatic Chronic Chagas Disease Patients Versus Healthy Subjects. Front Cell Infect Microbiol. 6 sept 2021;11:722984. |
| 10.1371/journal.pntd.0009874 | Casares-Marfil D, Kerick M, Andrés-León E, Bosch-Nicolau P, Molina I, Chagas Genetics CYTED Network, et al. GWAS loci associated with Chagas cardiomyopathy influences DNA methylation levels. PLoS Negl Trop Dis. oct 2021;15(10):e0009874. |
| 10.1186/s12920-021-01134-3 | Wu J, Cao J, Fan Y, Li C, Hu X. Comprehensive analysis of miRNA–mRNA regulatory network and potential drugs in chronic chagasic cardiomyopathy across human and mouse. BMC Med Genomics. déc 2021;14(1):283. |
| 10.3389/fimmu.2021.755782 | Teixeira PC, Ducret A, Langen H, Nogoceke E, Santos RHB, Silva Nunes JP, et al. Impairment of Multiple Mitochondrial Energy Metabolism Pathways in the Heart of Chagas Disease Cardiomyopathy Patients. Front Immunol. 12 nov 2021;12:755782. |
| 10.3389/fcvm.2021.751415 | Echeverría LE, Gómez-Ochoa SA, Rojas LZ, García-Rueda KA, López-Aldana P, Muka T, et al. Cardiovascular Biomarkers and Diastolic Dysfunction in Patients With Chronic Chagas Cardiomyopathy. Front Cardiovasc Med. 29 nov 2021;8:751415. |
| 10.1007/s10875-021-01000-y | Ouarhache M, Marquet S, Frade AF, Ferreira AM, Ianni B, Almeida RR, et al. Rare Pathogenic Variants in Mitochondrial and Inflammation-Associated Genes May Lead to Inflammatory Cardiomyopathy in Chagas Disease. J Clin Immunol. juill 2021;41(5):1048‑63. |
| 10.1016/j.parint.2021.102530 | Menezes TP, Machado BAA, Toledo DNM, Santos PV dos, Ribeiro L, Talvani A. Insights into CX3CL1/Fractalkine during experimental *Trypanosoma cruzi* infection. Parasitology International. avr 2022;87:102530. |
| 10.1016/j.imbio.2021.152166 | Torres DJL, Arruda TRD, Barros M da S, Gonçales JP, Soares AKA, Oliveira KK dos S, et al. Is a negative correlation between sTNFR1 and TNF in patients with chronic Chagas disease the key to clinical progression? Immunobiology. janv 2022;227(1):152166. |
| 10.1371/journal.pntd.0010074 | Arun A, Rayford KJ, Cooley A, Rana T, Rachakonda G, Villalta F, et al. Thrombospondin-1 expression and modulation of Wnt and hippo signaling pathways during the early phase of *Trypanosoma cruzi* infection of heart endothelial cells. Siqueira-Neto JL, éditeur. PLoS Negl Trop Dis. 5 janv 2022;16(1):e0010074. |
| 10.1155/2022/7641357 | do Carmo Neto JR, da Costa AWF, Braga YLL, Lucio FH, dos Santos Martins ALM, dos Reis MA, et al. The Colombian Strain of *Trypanosoma cruzi* Induces a Proinflammatory Profile, Neuronal Death, and Collagen Deposition in the Intestine of C57BL/6 Mice Both during the Acute and Early Chronic Phase. Bisoendial R, éditeur. Mediators of Inflammation. 12 janv 2022;2022:1‑9. |
| 10.1038/s41598-022-05493-4 | Farani PSG, Ferreira BIS, Gibaldi D, Lannes-Vieira J, Moreira OC. Modulation of miR-145-5p and miR-146b-5p levels is linked to reduced parasite load in H9C2 *Trypanosoma cruzi* infected cardiomyoblasts. Sci Rep. 26 janv 2022;12(1):1436. |
| 10.3389/fcimb.2022.836242 | Baron MA, Ferreira LRP, Teixeira PC, Moretti AIS, Santos RHB, Frade AF, et al. Matrix Metalloproteinase 2 and 9 Enzymatic Activities are Selectively Increased in the Myocardium of Chronic Chagas Disease Cardiomyopathy Patients: Role of TIMPs. Front Cell Infect Microbiol. 17 mars 2022;12:836242. |
| 10.3389/fimmu.2022.946350 | Grijalva A, Gallo Vaulet L, Agüero RN, Toledano A, Risso MG, Quarroz Braghini J, et al. Interleukin 10 Polymorphisms as Risk Factors for Progression to Chagas Disease Cardiomyopathy: A Case-Control Study and Meta-Analysis. Front Immunol. 4 juill 2022;13:946350. |
| 10.1016/j.imbio.2022.152242 | Silva MC, Fuzo CA, Marques Paiva I, Lopes Bibó N, Tavares de Oliveira M, da Silva Soares HA, et al. Synonymous mutation rs1129293 is associated with PIK3CG expression and PI3Kγ activation in patients with chronic Chagas cardiomyopathy. Immunobiology. sept 2022;227(5):152242. |
| 10.3389/fcimb.2022.890709 | Lopez JR, Linares N, Adams JA, Mijares A. The Role of the Na+/Ca2+ Exchanger in Aberrant Intracellular Ca2+ in Cardiomyocytes of Chagas-Infected Rodents. Front Cell Infect Microbiol. 7 juill 2022;12:890709. |
| 10.1016/j.actatropica.2022.106651 | Medina L, Guerrero-Muñoz J, Castillo C, Liempi A, Fernández-Moya A, Araneda S, et al. Differential microRNAs expression during ex vivo infection of canine and ovine placental explants with *Trypanosoma cruzi* and Toxoplasma gondii. Acta Tropica. nov 2022;235:106651. |
| 10.3390/microorganisms10081602 | Rodrigues AB, da Gama Torres HO, Nunes M do CP, de Assis Silva Gomes J, Rodrigues AB, Pinho LLN, et al. Biomakers in Chronic Chagas Cardiomyopathy. Microorganisms. 9 août 2022;10(8):1602. |
| 10.3389/fimmu.2022.958200 | Brochet P, Ianni BM, Laugier L, Frade AF, Silva Nunes JP, Teixeira PC, et al. Epigenetic regulation of transcription factor binding motifs promotes Th1 response in Chagas disease cardiomyopathy. Front Immunol. 22 août 2022;13:958200. |
| 10.3390/biomedicines10092215 | Silva KDA, Nunes JPS, Andrieux P, Brochet P, Almeida RR, Kuramoto Takara ACK, et al. Chagas Disease Megaesophagus Patients Carrying Variant MRPS18B P260A Display Nitro-Oxidative Stress and Mitochondrial Dysfunction in Response to IFN-γ Stimulus. Biomedicines. 7 sept 2022;10(9):2215. |
| 10.3390/ijms231810456 | Díaz ML, Burgess K, Burchmore R, Gómez MA, Gómez-Ochoa SA, Echeverría LE, et al. Metabolomic Profiling of End-Stage Heart Failure Secondary to Chronic Chagas Cardiomyopathy. IJMS. 9 sept 2022;23(18):10456. |
| 10.1080/08820139.2022.2110503 | Oliveira Cavalcanti E, Freitas Lidani KC, de Freitas Oliveira Toré C, de Messias Reason IJ, Andrade FA. MASP1 Gene Polymorphism and MASP-3 Serum Levels in Patients with Chronic Chagas Disease. Immunol Invest. oct 2022;51(7):2108‑21. |
| 10.1371/journal.pntd.0010725 | Sabino EC, Franco LAM, Venturini G, Velho Rodrigues M, Marques E, Oliveira-da Silva LC de, et al. Genome-wide association study for Chagas Cardiomyopathy identify a new risk locus on chromosome 18 associated with an immune-related protein and transcriptional signature. Almeida IC, éditeur. PLoS Negl Trop Dis. 10 oct 2022;16(10):e0010725. |
