# Supplementary material for: ChagasDB: 80 years of publicly available data on the molecular host response to Trypanosoma cruzi infection in a single database
Source: Database (Oxford). 2023 May 26;2023:baad037. doi: 10.1093/database/baad037 (PMC10205463; doi:10.1093/database/baad037)
Supplement: baad037_Supp [file baad037_supp.zip › suppl_data/Supplementary_Table 3.docx]

**Supplementary table 3.** Number of features and papers dealing with each phenotype appearing in the ChagasDB.

|  | **Phenotype** | **Number of features per phenotype and per paper** | **Number of paper per phenotype** |
| --- | --- | --- | --- |
| **Non-infected phenotypes** | Healthy | 67366 | 149 |
|  | Non chagasic cardiomyopathy | 44 | 4 |
|  | Non chagasic dilated cardiomyopathy | 12 | 4 |
|  | Idiopathic dilated cardiomyopathy | 11 | 2 |
|  | Ischemic cardiomyopathy | 11 | 1 |
| ***T.cruzi* infected phenotype** | Chagas | 173 | 41 |
|  | Acute | 43938 | 51 |
|  | Chronic | 4805 | 22 |
|  | Asymptomatic | 12789 | 43 |
|  | Indeterminate | 167 | 34 |
|  | Symptomatic | 33 | 8 |
|  | Cardiac | 35336 | 86 |
|  | Moderate cardiac | 6842 | 21 |
|  | Mild cardiac | 27 | 7 |
|  | Severe cardiac | 16096 | 27 |
|  | Digestive | 43 | 15 |
|  | Cardiodigestive | 18 | 5 |
|  | Mixed | 24 | 3 |
|  | Congenital infection | 5 | 1 |
|  | Heart transplant chagasic patient | 5 | 1 |
